# Agroforestry diversity, indigenous food consumption and nutritional outcomes in Sauria Paharia tribal women of Jharkhand, India

**DOI:** 10.1111/mcn.13052

**Published:** 2020-07-27

**Authors:** Suparna Ghosh‐Jerath, Ridhima Kapoor, Archna Singh, Nilima, Shauna Downs, Gail Goldberg, Jessica Fanzo

**Affiliations:** ^1^ Indian Institute of Public Health‐Delhi Public Health Foundation of India Gurgaon India; ^2^ Department of Biochemistry All India Institute of Medical Sciences New Delhi India; ^3^ Department of Biostatistics All India Institute of Medical Sciences New Delhi India; ^4^ Department of Urban‐Global Public Health Rutgers School of Public Health Newark New Jersey USA; ^5^ Nutrition and Bone Health Research Group, MRC Human Nutrition Research Elsie Widdowson Laboratory Cambridge UK; ^6^ Berman Institute of Bioethics, Johns Hopkins Nitze School of Advanced International Studies, Department of International Health Johns Hopkins Bloomberg School of Public Health Baltimore Maryland USA

**Keywords:** dietary diversity, indigenous foods

## Abstract

Like several indigenous populations, Sauria Paharias, a vulnerable indigenous tribal group residing in a biodiverse environment of Jharkhand, India, have high levels of undernutrition. We assessed agroforestry and dietary diversity, food consumption especially indigenous food (IF) intake and nutritional status of Sauria Paharia women through a cross‐sectional study conducted in 18 villages of Godda district, Jharkhand. Household level information was elicited through household surveys including a dietary survey and a food frequency questionnaire. Twenty‐four‐hour dietary recalls (24 HDR) and anthropometric assessments were taken on one randomly selected woman per household. An index, Food Accessed Diversity Index (FADI) created to measure agroforestry diversity, showed a low mean score of 0.21 ± 0.15 and range: 0, 0.85. Fifty‐nine percent of women consumed any IF during 24 HDR. Median minimum dietary diversity score for women (MDD‐W) was 3 (acceptable score ≥5). More than 96% of women had intakes below estimated average requirements for all nutrients studied (energy; vitamins A, C, thiamine, riboflavin, niacin, pyridoxine; folate; iron; calcium and zinc) except protein; 41% women were underweight. IF consumption was independently associated with calcium and vitamin A intake. Decision trees developed for micronutrient consumption at different levels of MDD‐W score and IF consumption scenarios revealed 1.3 to 2.9 times higher consumption of micronutrients among women with MDD‐W ≥ 3 or 4. Strategies like agricultural extension programmes promoting indigenous varieties and nutrition education for increasing dietary diversity with IFs have potential to address undernutrition in Sauria Paharia women.

**Key messages**

- Access to agroforestry diversity was poor among Sauria Paharias despite extensive collective information on availability of diverse traditional foods in their environment.
- Women had poor nutrient intake and dietary diversity.
- IF consumers had significantly higher intake of calcium and vitamin A.
- Stratification by MDD‐W scores and IF consumption revealed 1.3 to 2.9 times higher consumption of different micronutrients among women with MDD‐W equal to or above 3 or 4 and IF consumers.
- Access to agroforestry diversity can be improved through agricultural extension programmes to promote cultivation of indigenous varieties and nutrition education for improving dietary diversity utilizing IFs from the environment.

## 1 INTRODUCTION

Globally, more than 820 million people are undernourished, and more than 2 billion people suffer from micronutrient deficiencies (FAO, 2017). More than one fifth of women in India are undernourished with chronic energy deficiency (CED), and more than 50% of them have anaemia (National Family Health Survey [NFHS]‐4, 2015). The country is ranked 102nd among 117 nations on the global hunger index, and its status is rated as “serious” on the spectrum of hunger levels (Global Hunger Index [GHI], 2019). Moreover, India is ranked 70th among 187 countries on dietary quality, based on overall dietary patterns (Imamura et al., 2015), with diets that are less diverse, deficient in dairy, fruits, vegetables and protein rich foods (Keats & Wiggins, 2014). Both insufficient quantity and quality of food intake are causing food and nutrition insecurity leading to malnutrition within the country.

Indian agriculture, boosted by the Green Revolution in the mid‐60s, mainly focuses on wheat and rice production, with a goal of fulfilling average caloric requirements (Dwivedi et al., 2017; Welthungerlife, 2016). Additionally, the targeted public distribution system, a major government programme under the national food security act (NFSA, 2013), mainly includes food items like wheat, rice, sugar and millets (in specific states). This strategy has promoted poor quality, monotonous and low‐diversity diets, especially among vulnerable sections of the population (Welthungerlife, 2016). More recently, there has been increased effort towards promoting the cultivation and distribution of diverse cereals like millets through this programme (Re‐Vamped National Food Security Mission [NFSM] Operational Guidelines [2018–2019 to 2019–2020], 2018).

Indigenous tribal people are those who are original or earliest known inhabitants of an area and inherit their ancestral knowledge of the land and food resources, available in the vicinity of their residence (Kuhnlein et al.,, 2013). These communities, referred to as Scheduled tribes and as described by Article 342 of Indian Constitution, are distinguished from other communities based on their traditional traits, distinct culture and economic backwardness (Tribal Health in India: Executive Summary and Recommendations, 2018). They constitute a substantial proportion of the nutritionally vulnerable population of India with women having high prevalence of CED and micronutrient deficiencies and under five children having high levels of stunting (40%), underweight (40%) and wasting(27%) (Agrawal, 2013; Ghosh‐Jerath et al., 2013, 2016; Kshatriya & Acharya, 2016; National Nutrition Monitoring Bureau [NNMB], 2009; National Family Health Survey [NFHS]‐4, 2015). The diets of Indian tribal communities are considerably deficient in milk and milk products, fruits and vegetables, as well as pulses and flesh foods (meat, fish and poultry) (Ghosh‐Jerath et al., 2016; Mittal & Srivastava, 2006; Rao, Kumar, Krishna, Bhaskar, & Laxmaiah, 2015). This predisposes them to deficiencies of micronutrients like iron, vitamin A (Rao et al., 2015) and also zinc, which could be due to the high phytate content in staples like rice (Kapil, Singh, & Pathak, 2003). This nutritional deprivation is further compounded by socio‐ecological factors such as geographical isolation, extreme poverty, migration, local agricultural and land use policies and climate change (Bhattacharjee, Kothari, Priya, & Nandi, 2009; Jain et al., 2015; Laxmaiah et al., 2007; Rao et al., 2015).

The Sauria Paharias are one of the particularly vulnerable tribal groups (PVTGs) in Jharkhand, a state that is home to 8.6 million indigenous tribal people constituting 26.2% of the total state population (Census, 2011; Lakra & Kumar, 2017). The Sauria Paharias reside among the hilly ranges and wild dense forests, which provides a rich biodiverse food environment (UNDP, 2008). An ancient tribal group with predominantly agriculture‐based livelihoods, they practise cultivation on small areas of land, home gardens (*Baaris*) and burnt patches of forest land (*Kurwa* farming). They also source foods from forests, rivers and nearby areas that are indigenous to the community, utilizing these either for household consumption or income generation (Tribal Cultural Heritage in India Foundation, 2018). However, despite a rich biodiverse agroforestry environment, the Sauria Paharias are impoverished and lag behind on all nutrition indicators (Kumar & Sinha, 2016).

Food‐based strategies such as incorporating traditional and indigenous foods (IFs) obtained locally from the natural environment through farming and wild harvesting can potentially improve dietary diversity and micronutrient deficiencies (Dwivedi et al., 2017; Sethi et al., 2017). Studies have shown a positive impact of production diversity on child anthropometric outcomes (Jones, 2017); better access to diverse and improved diets through home gardening and consumption of indigenous varieties of fruits and vegetables from forests and other nearby areas could likely improve micronutrient status (Berti, Krasevec, & FitzGerald, 2004; Girard, Self, McAuliffe, & Olude, 2012; Masset, Haddad, Cornelius, & Isaza‐Castro, 2012; Tontisirin, Nantel, & Bhattacharjee, 2002). Coincidently, low‐income regions with high levels of malnutrition are often rich biodiversity hotspots with nutrient resources that are often underutilized (Herrero et al., 2017; Lachat et al., 2018; Myers, Mittermeier, Mittermeier, da Fonseca, & Kent, 2000). Therefore, leveraging this biodiversity (‘Agroforestry and its Benefits|Biodiversity’; Palacios Bucheli & Bokelmann, 2017) to improve access and utilization of traditional foods can facilitate consumption of diverse, affordable diets among the poorer and indigenous communities (Jones, 2017; Lachat et al., 2018). Because the relationship between agroforestry diversity, actual dietary intake and nutritional outcomes is affected by multiple factors (Gómez et al., 2013; Pinstrup‐Andersen, 2013), a detailed enquiry can help identify the pivotal determinants affecting this relationship under specific contexts. An integrated approach built on these findings can strengthen and make modern agriculture nutrition sensitive by mainstreaming the traditional food systems into it (Jones, 2017; Welthungerlife, 2016).

In this paper, we explore the association between production and access to diverse foods (agroforestry diversity), IF consumption and dietary diversity with nutrient intake in the Sauria Paharia community. We also analyse the impact of external factors such as socio‐economic and demographic profile on this relationship. Finally, we explored quantitative differences in mean micronutrient intakes stratified both by dietary diversity and IF consumption.

## 2 METHODS

This study was part of a larger project that examined IF consumption by tribal groups of Jharkhand and its contribution to dietary diversity and food security among women and children. A detailed description of the larger project and its methodological approaches are reported elsewhere (Ghosh‐Jerath et al., 2019). In the present paper, we report the socio‐economic, demographic, dietary profile, agroforestry diversity, IF consumption, nutrient intake and nutritional status of Sauria Paharia women. The exploratory cross‐sectional survey was conducted in July–August 2018, during the rainy season, in Sauria Paharia villages.

### 2.1 Study area

The study was conducted in selected Sauria Paharia villages in two blocks of Godda district of Jharkhand (Sauria Paharia population in Godda district = 13,688; Census, 2011).

### 2.2 Sampling framework and study population

**FIGURE 1:**
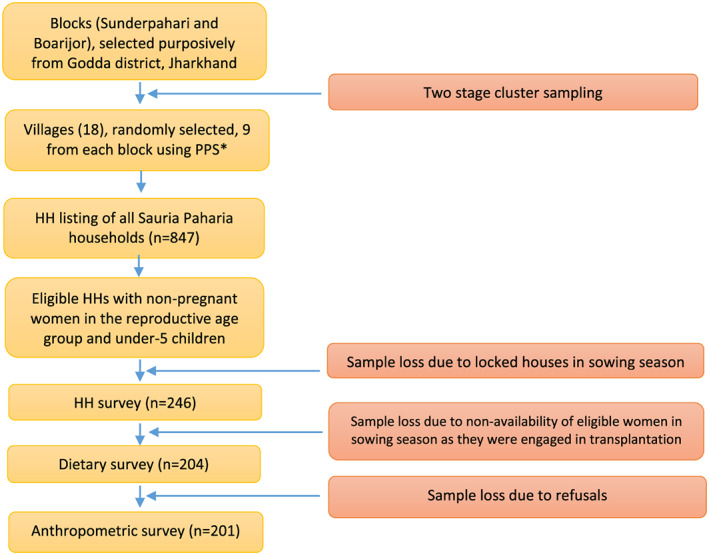
Flow diagram depicting data collection. *PPS‐Probability Proportional to Size Sampling

A two‐stage cluster sampling design was followed (Figure 1). From the purposively chosen two blocks (a block is defined as an administrative subdivision of a district), namely, Sunderpahari and Boarijor, and by using a tribal village list, in the first stage, nine villages each from the blocks were randomly selected using probability proportional to size (PPS) sampling. In the second stage, all 18 selected villages were visited, and a house‐listing exercise for all Sauria Paharia households (HHs) was carried out to construct the sampling frame of all eligible HHs. The eligibility was based on the overall objective and presence of at least one non‐pregnant woman in the reproductive age group (15–49 years) and one child (6 months to 54 months) in the HH. In case of more than one eligible woman in a HH, one woman was randomly selected using Kish table (WHO World Health Survey, 2002). Two more subsequent visits were done to complete the HH survey, 2 days' dietary recall and anthropometric assessment.

### 2.3 Sample size calculation

Requisite sample size was calculated based on the difference in mean dietary intake of iron of 4 mg/day (35% increase) with a standard deviation (SD) of 7 mg/day between consumers and non‐consumers of IFs, reported in a previous study (Ghosh‐Jerath et al., 2016) among women of Santhal tribes. A minimum of 97 women in each group were required to be sampled to detect the suggested minimum difference in iron intake across the two groups with 80% power and a 5% level of significance. A design effect of 2.0 was considered to account for the loss in precision due to cluster sampling and deviation from normality. The above calculation suggested a sample size of 194 women. In order to get these 194 HHs in the study villages, a HH listing was done to cover all HHs (*n* = 847) in 18 villages, and all eligible HHs were selected (*n* = 295). HH surveys could be completed in 247 out of 295 HHs, and dietary and anthropometric assessments were completed in 204 and 201 women respectively.

### 2.4 Data collection

A pretested questionnaire developed on an electronic data capture (EDC) platform using Samsung (Model SM‐T385) and the software CS pro (Version 7.1) was administered at the HH level to elicit information on socio‐economic and demographic profile. Information on availability of and access to different food sources was collected using HH survey tool and agriculture diversity tool. The software had extensive in‐built checks (context, range and logic checks). Frequency of intake of different foods at HH level with a reference period of 1 month was assessed using a food frequency questionnaire (FFQ). A 24‐h dietary recall (24 HDR) and anthropometric assessment on one selected woman was also done. Paper forms were used for FFQ and 24 HDR and administered by nutritionists and nutrition interns after due training. The field investigators administered the HH questionnaire on EDC platform and did anthropometric assessments after due trainings and retraining on need basis. Standard procedures were followed for anthropometric assessments, and routine back‐end data checks were conducted to maintain data quality.

### 2.5 Study variables

The outcome variables for this study included intake of macronutrient, that is, energy (Kcal), protein (g), fats (g) and micronutrients including iron (mg); calcium (mg); zinc (mg); vitamin A (μg); vitamins B1, B2, B3 and B6 in (mg); folic acid (μg) and vitamin C (mg) and anthropometric status of women. The exposure variables included IF consumption at the individual level, minimum diet diversity for women of reproductive age (MDD‐W; a validated measure of dietary diversity; Minimum Dietary Diversity for Women‐ A Guide to Measurement, 2016) and an index developed for agroforestrydiversity at HH level. Other independent variables included socio‐demographic profile of the HHs, HH wealth index and access to different sources of food like agricultural land, forests and water sources. A brief description of each these variables is given as follows:

Measurement of nutrient intake: A nonconsecutive, 2‐day multiple pass 24 HDR method was used for nutrient intake estimation of one selected woman per HH. The selected women were asked to recall all food items consumed over the past 24 h and show (wherever possible) or describe the foods eaten in each meal (i.e., each food item consumed along with a detailed recall of ingredients used in preparation and method of preparation). Food recall kit (standard utensils and food picture flip book) was used for portion size estimation. At the end, a final probing was done to elicit information of foods consumed but inadvertently forgotten. Self‐reported amounts of raw foods were used to determine the daily intake of individual food items belonging to the different food groups such as cereals, pulses, flesh foods, green leafy vegetables (GLVs), other vegetables, roots and tubers, fruits, sugars and fats.

Measurement of dietary diversity: Dietary diversity for women was measured as per FAO and USAID guidelines for MDD‐W (Minimum Dietary Diversity for Women‐ A Guide to Measurement, 2016). The MDD‐W was calculated by adding the number of food groups (for food items with amounts ≥15 g) consumed by the women as reported in the 24 HDR. Women who consumed ≥5 food groups were considered to be meeting recommendations for minimum dietary diversity.

#### 2.5.1 Consumption of different foods at household level

The frequency of consumption of different food items under various food groups at HH level was assessed using FFQ. Food items identified during FGDs conducted in the same community in previous surveys were extensively used to develop a FFQ including both commonly consumed Indian food items and Indigenous tribal foods. We developed and pretested a 300‐item FFQ with the community. A list of IFs reported by the community along with their taxonomic classification (wherever available) is provided as Table S1.

#### 2.5.2 IF consumption

This variable was calculated from the list of food items reported in the 24 HDR. If a woman consumed any IF (as listed in table S1) over the recall period of 2 days, she was considered as a consumer of IF.

#### 2.5.3 Nutritional status

Anthropometric measurements (heights and weights) were taken to assess the nutritional status of the women from whom DRs were taken. These measurements were carried out using standard protocols and equipments that included ‘Seca’ digital flat scale Model 813 for weight measurement and ‘Seca’ stadiometer Model 213 for height measurement.

#### 2.5.4 Socio‐demographic characteristics

A pretested structured questionnaire was developed to elicit information on HH members' characteristics, which included family type, age profile of family members and educational and occupational status of family members (selected women participant and head of the household [HOH]). HH information like type of roofing material, number of rooms, presence of a separate kitchen, source of electricity and drinking water, possession of assets (cot, chair, kerosene stove and mobile phone) and monthly expense on food were elicited and used to generate the HH wealth index variable.

#### 2.5.5 Access to food sources

To elicit information on food access during the past 1 year, self‐reported information on HHs ownership of agricultural land and kitchen garden (baari), access to forest land for “Kurwa farming” and for food gathering and access to water bodies were collected.

#### 2.5.6 Measurement of agroforestry diversity

Information of total number of foods accessed from different sources (as mentioned above) was obtained through HH questionnaire and an agricultural diversity tool. The latter was administered on conveniently selected 60 HHs.

In previous studies, agricultural diversity has been simply calculated by the total number of food groups grown and/or types of animals raised for food (Hirvonen & Hoddinott, 2016; K. T. Sibhatu & Qaim, 2016). In our study, we modified the agroforestry diversity component by constructing an index called Food Accessed Diversity Index (FADI), which has been adapted from crop diversity index (CDI) (Michler & Josephson, 2017). We divided the total number of foods grown, gathered or accessed as well as animals raised in a particular HH by the corresponding maximum possible number of foods grown, gathered, accessed and raised in a particular village (*N*). Instead of standard number of food groups, we preferred to do counts of individual food items. This is because nutritive value of several food items under a specific food group may vary a lot. The FADI was expressed as

$$\text{FADI}={\left(\frac{n}{N}\right)}^{2}.$$

Thus, FADI is a relative expression. Foods accessed from the market were not included in this index. Lower values of FADI indicate lower diversity in production and access to foods and vice versa. This index helped in capturing the inequality in production and access between HHs of the same village, while adjusting for agro‐climatic conditions, specific to a village (Michler & Josephson, 2017).

### 2.6 Data analysis

Quantitative variables were reported as Mean ± SD, and qualitative variables were summarized using counts and percentages. Outliers, if any, were retained unless there was a data entry mistake. The information on number of food sources accessed were converted to scores. HH wealth index score was obtained using the principal component analysis (PCA), an adaptive statistical technique for reducing the dimensionality of large data sets. The HHs were then classified into five groups based on the quintiles of the HH wealth index score. MDD‐W was computed by taking median of total food groups consumed over 2 days. MDD‐W was later classified into two levels *less than median* and *more than or equal to median.* BMI was calculated as weight (in kg) divided by height (in m^2^) based on standard practices. The women were then classified as being underweight or, otherwise, using the BMI cut‐offs. The women were further classified under different levels of CED using the standard BMI cut‐offs (WHO Expert Consultation, 2004).

The food intake data from 2 days 24 HDR were converted to nutrient intakes using a validated software ‘DietCal’ (Version 8.0; Profound Tech Solution), which is based on values from Indian Food Composition Table, 2017 (Longvah, Ananthan, Bhaskarachary, & Venkaiah, 2017). Because most of the nutrient intakes were non‐normally distributed, the box‐cox method was used. Box‐cox parameter lambda was observed to be zero, and hence, a log transformation was considered to be most appropriate for all nutrient intakes including iron. The transformed 2‐day 24 HDR data on macronutrient and micronutrient intakes were then used in linear mixed effect regression (LMER) model. The values predicted using the LMER model were back‐transformed to compute the ‘usual nutrient intake’ (Tooze et al., 2010). These usual intakes were utilized for estimating the prevalence of nutrient adequacy using estimated average requirement (EAR) cut‐point method (Dietary Reference Intakes, 2000) for all nutrients, except iron. For iron, the full probability approach was used for estimating adequacy (Dietary Reference Intakes, 2000). The EAR for all nutrients, except for energy, protein and iron, was calculated by multiplying the Recommended Dietary Allowances (RDA) for moderately active women and adolescent girls (wherever applicable; ICMR, 2010) by a factor of 0.77 (Gibson, 2008). Details are provided in Table 2. The usual intake between IF consumers and nonconsumers and MDD‐W below or above median were compared using independent samples *t*‐test.

To account for the nested structure of the data, LMER technique with villages (*n* = 18) as the random effect was adopted, to investigate linear relationship of usual intake of each nutrient with IF consumption and MDD‐W adjusting for other factors (presented in Table 4). The usual nutrient intakes obtained were also observed to be skewed and hence were again transformed using box‐cox procedure as illustrated above. The hence obtained usual nutrient intakes were used for further analysis. Here, each log transformed usual nutrient intake was considered as an outcome variable. Multicollinearity was assessed. The associated factors under investigation were MDD‐W, IF consumption status, FADI, HH wealth index, education status of selected women and HOH. The education, MDD‐W, IF consumption and the HH wealth index were treated as categorical variables. Reference category for each categorical variable and the Intraclass correlation coefficient are reported in Table 4. A *P*‐value of less than or equal to 0.05 was considered to be significant throughout. The data analysis was performed using R software.

A nutrient intake decision tree was developed for micronutrients to identify the best possible path to improve the nutritional intake under different scenarios of MDD‐W scores and IF consumption versus nonconsumption among the study population. A decision tree is a flowchart like arrangement of decisions and their possible consequences. This visual display of algorithm contains conditional control statements to search for possible variables and values in order to find the best split.

### 2.7 Ethical considerations

The study protocol was approved by the Institutional Ethics Committee at Indian Institute of Public Health‐Delhi, Public Foundation of India and All India Institute of Medical Sciences, New Delhi. Administrative approvals from authorities at district level was also taken. Cluster level consent from the village leader was obtained before any data collection. All participants provided informed consent prior tothe commencement of the interviews. Verbal witnessed consent was taken from illiterate participants in presence of third party; signedconsent was taken from literate participants. Participation in the study was voluntary and small gifts, procured from local markets were given as incentives to the selected woman of the HH.

## 3 RESULTS

### 3.1 Household profile

A total of 246 Sauria Paharia HHs with a population of 1,492 inhabiting 18 villages of two blocks of Godda district of Jharkhand were surveyed. Table 1 provides the socio‐demographic and economic profile of the HHs interviewed. Adult women comprised about 21.4% of the total population. Mean age of non‐pregnant women in the reproductive age group (15–49 years) was 27 ± 8 years. Majority of the HHs (91.2%) had male members as HOH, and about 62.2% HHs were nuclear families. Most of the HHs (78.1%) were engaged in agriculture as their primary occupation. The population was classified into quintiles of HH wealth index score with a mean score of −2.19 ± 0.74 and −0.8 ± 0.25 for the lowest two quantiles.

**TABLE 1:** Socio‐demographic and economic profile of the Sauria Paharia households (*N* = 246)

Characteristics	*n* (%)
Family type	
Nuclear	153 (62.2)
Joint	90 (36.6)
Extended	3 (1.2)
Religion	
Christian	127 (51.6)
Hindu	108 (43.9)
Others	11 (4.5)
Gender of HOH	
Male	227 (92.3)
Female	19 (7.7)
Literacy level of HOH	
No formal education	97 (39.5)
No formal education but can sign	23 (9.4)
No formal education but can read and write	2 (0.8)
Less than primary (till 4th standard)	22 (8.9)
Primary but less than secondary (till 9th standard)	80 (32.5)
Secondary (10th standard) and above	22 (8.9)
Occupation of HOH	
Settled agriculture/shifting cultivation	192 (78.1)
Daily wager (agriculture and non‐agriculture)	37 (15.1)
Hunter/gatherer	5 (2)
Craftsmen/artisans/self‐employed	3 (1.2)
Service (government and private)	6 (2.4)
Unemployed	2 (0.8)
Housewife	9 (0.4)
Literacy level of selected women	
No formal education	151 (61.4)
No formal education but can sign	26 (10.6)
No formal education but can read and write	2 (0.8)
Less than primary (till 4th standard)	19 (7.7)
Primary but less than secondary (till 9th standard)	43 (17.5)
Secondary (10th standard) and above	5 (2.1)
PDS ration card^a^	
Yellow (AAY)	153 (62.2)
Red (BPL)	40 (16.3)
Do not have	53 (21.5)
Characteristics	Mean (SD)
Age of selected women in years Mean ± SD	26.8 ± 7.87
Number of family members Mean ± SD	6.1 ± 1.96
Age of the eldest member in the HH in years Mean ± SD	40.4 ± 13.91
HH wealth index Mean ± SD	
Lowest quintile	−2.19 ± 0.74
Lowest middle quintile	−0.8 ± 0.25
Lower middle quintile	0.02 ± 0.23
Upper middle quintile	0.81 ± 0.23
Upper most quintile	1.79 ± 0.39

Abbreviations: HOH, head of the household; SD, standard deviation; AAY, Antyodaya Anna Yojana; APL, Above Poverty Line; BPL, Below Poverty Line

^a^ PDS refers to Public Distribution system which is an Indian food security scheme under Ministry of Consumer Affairs, Food and Public Distribution. In this, major food commodities including staple food grains, such as wheat and/or rice, sugar and kerosene oil (a fuel used for cooking) are distributed through a network of public distribution shops (also known as ration shops) at subsidized prices. Possession of a PDS ration card (an official document entitling the holder to a ration of food) under various categories of poverty, that is, APL , BPL and AAY, a category based on degrees of poverty, entitles the holder to access the food product at highly subsidized rates.

### 3.2 Availability and access to food sources

The community accessed agricultural land, kitchen gardens and burnt patches of forest for farming and raised livestock to produce home grown foods. They also accessed forest, water bodies (ponds/rivers) and markets to collect and purchase food. Most HHs owned agricultural land and kitchen gardens (90.2%), and almost all (96.5%) were using agricultural produce for HH consumption. The majority reported accessing forests (93.5%) and water bodies (83.3%) for collecting food items such as fruits, vegetables, roots and tubers, mushrooms, fishes and wild animals for HH consumption. More than half of the HHs (60%) owned livestock such as goat, sheep, cattle, poultry and pigs, and 84.5% of them used these for HH consumption.

Despite a majority of HHs accessing agriculture land, forests, ponds, rivers and livestock, a low mean FADI of 0.21 ± 0.15 was obtained for the Sauria Paharia community (range: 0, 0.85). This low FADI score depicts poor diversity in the context of food availability and access. The mean of total number of food items available per village without considering the foods bought from the market was 24.5 ± 4.56 (range:13, 31).

### 3.3 Dietary intake pattern of adults in the HHs

Based on the FFQ, most (45.8%) HHs consumed rice as a staple food twice a day during the previous month. Pulses along with roots and tubers constituted the other food items in the habitual diets of the community. Almost half (46.6%) the population reported consuming roots and tubers twice a day. About 68.4% and 65.8% of the HHs consumed pulses and GLVs three or more times a week, respectively, and approximately 55% of the HHs also consumed other vegetables once or twice a week. The majority of the HHs (55.8%) reported consuming flesh foods once or twice a week. Daily consumption of fruits was reported by more than half (56.7%) of the HHs. The majority (80.8%) of the HHs did not report consuming any milk and milk products. Sugar consumption was reported once a day by one third of HHs. Consumption of mustard oil was reported by most (90.1%) of the HHs with 88.3% of them consuming it for two or more times daily. Consumption of different indigenous varieties of alcohol drinks was reported, which included *mahua taadi* (alcoholic drink made from mahua), *khajur taadi* (alcoholic drink made from dates) and *handiya* (alcoholic drink made from rice). About 43.3% of the HHs reported consuming these alcoholic beverages once or twice a week.

### 3.4 Nutrient intake of women in the reproductive age group (15–49 years)

Table 2 provides a detailed description of women's nutrient intake based on the 24 HDR. The mean intakes for all nutrients based on usual intakes of study participants were less than the EAR values in adult women and adolescent girls (16–17 years old). However, among adolescent girls (15 years of age), mean intakes were less than EAR for all nutrients except protein. More than 96% of women of all age groups had intakes of energy, calcium, iron, zinc, vitamin A, vitamin C, thiamine, riboflavin, niacin, pyridoxine and folate below EAR.

**TABLE 2:** Mean of usual nutrient intakes and prevalence of nutritional inadequacy in study population (*N* = 204)

Nutrients	Women in the age group:18–49 years, *n* = 192		Women in the age group: 16–17 years, *n* = 7		Women in the age group: 15 years, *n* = 5		*n* (%) < EAR (compiled *N* = 204)
	EAR	Mean Intake ± SD	EAR	Mean Intake ± SD	EAR	Mean Intake ± SD	
Energy (kcal/d)	2230^a^	1,093 ± 95	2440^a^	1,035 ± 92	2330^a^	1,148 ± 72	204 (100)
Protein (g/d)	35.7^a^	31.4 ± 4.3	33.9^a^	28.8 ± 3.2	30.3^a^	34.6 ± 3	174 (85.2)
Fat (g/d)	NA^c^	8 ± 1.5	NA^c^	7.1 ± 1.3	NA^c^	9.1 ± 1.5	NA
Calcium (mg/d)	462^b^	95.2 ± 28.7	616^b^	81.9 ± 16.5	616^b^	94.8 ± 12.9	204 (100)
Iron (mg/d)	15^d^	5.4 ± 1.6	NA	4.7 ± 1.3	NA	5.5 ± 1.5	204 (100)^*^
Zinc (mg/d)	7.7^b^	4.5 ± 0.7	9.2^b^	4.1 ± 0.6	8.5^b^	4.7 ± 0.6	204 (100)
Vitamin C (mg/d)	30.8^b^	13.9 ± 8.1	30.8^b^	10.9 ± 10.1	30.8^b^	12.5 ± 5.2	196 (96.1)
Vitamin A (μg/d)	462^b^	30.8 ± 68.1	462^b^	9 ± 8.2	462^b^	7.2 ± 6.5	203 (99.5)
Thiamine (mg/d)	0.8^b^	0.3 ± 0.1	0.8^b^	0.3 ± 0.1	0.9^b^	0.4 ± 0.1	203 (99.5)
Riboflavin (mg/d)	1^b^	0.2 ± 0.1	0.9^b^	0.2 ± 0.04	1.1^b^	0.2 ± 0.05	204 (100)
Niacin (mg/d)	10.8^b^	5.4 ± 0.8	10.8^b^	4.9 ± 0.6	10.8^b^	5.5 ± 0.6	204 (100)
Pyridoxine (mg/d)	1.5^b^	0.5 ± 0.1	1.5^b^	0.5 ± 0.1	1.5^b^	0.5 ± 0.1	204 (100)
Folate (μg/d)	154^b^	81.6 ± 24.5	154^b^	65.9 ± 20.8	115^b^	90.7 ± 23.4	200 (98.04)

Abbreviations: NA, not available; EAR, estimated average requirements; SD, Standard Deviation

^a^ EAR value available in the Recommended Dietary Allowances for Indians (ICMR, 2010).

^b^ EAR calculated after multiplying RDA by a factor of 0.77 (Gibson, 2008).

^c^ EAR not available for fat.

^d^ Ghosh, Sinha, Thomas, Sachdev, and Kurpad (2019).

^*^ Prevalence of iron inadequacy using probability approach method.

### 3.5 Dietary diversity and IF consumption and their contribution to nutrient intake of women (15–49 years)

Based on the FFQ data, around 30% of the HHs reported twice‐a‐day consumption of indigenous varieties of cereals, and about half of them reported consuming indigenous pulses at least once a week. Similar weekly consumption was reported for indigenous varieties of fruits (30%), GLVs (40.8%), other vegetables (35.8%), flesh foods (43.3%) and roots and tubers (30%). About 40% of HHs reported consuming indigenous mushrooms at least once a month.

To explore the contribution of IFs to nutrient intake, we compared the 24 HDR data in women who consumed IFs in the past 2 days with the women who did not (Table 3). Around 59% of the study population (*n* = 204) consumed at least one IF in one of the recall days. Those who consumed IFs reported median intake of indigenous cereals as 248 g, pulses as 45 g, GLVs as 43 g, other vegetables as 16 g, fruits as 38 g and roots and tubers as 74 g, respectively. At these consumption levels of IFs, the intakes of riboflavin, calcium and vitamin A were found to be significantly higher among IF consumers. The mean MDD‐W for the study population was found to be very low, with the median of 3 (range: 1, 5). Women with MDD‐W scores higher than or equal to 3 had a significantly higher intake for all the nutrients. No significant association was observed between the HH Wealth Index and MDD‐W scores (*P* = 0.198).

**TABLE 3:** Comparisons of mean usual intakes between the IF consumers and categories of MDD‐W (*N* = 204)

Nutrient	IF consumers	Non‐IF consumers	*P‐* value	MDD‐W ≥ 3	MDD‐W < 3	*P*‐value
	Mean ± SD	Mean ± SD		Mean ± SD	Mean ± SD	
Energy (kcal/d)	1,089 ± 103	1,096 ± 85	0.583	1,113 ± 84	1,056 ± 103	**<0.001**
Protein (g/d)	31.3 ± 4.6	31.5 ± 3.9	0.796	32.4 ± 4.1	29.7 ± 4.3	**<0.001**
Fat (g/d)	7.9 ± 1.5	8.1 ± 1.5	0.499	8.4 ± 1.5	7.4 ± 1.2	**<0.001**
Calcium (mg/d)	100.6 ± 31.2	86.3 ± 20.9	**<0.001**	100.7 ± 28.3	84.5 ± 25.3	**<0.001**
Iron (mg/d)	5.4 ± 1.6	5.4 ± 1.4	0.917	5.7 ± 1.6	4.9 ± 1.4	**<0.001**
Zinc (mg/d)	4.4 ± 0.8	4.5 ± 0.7	0.321	4.6 ± 0.7	4.2 ± 0.7	**<0.001**
Vitamin C (mg/d)	14.2 ± 8.7	13.2 ± 7.2	0.391	15.2 ± 8.2	11.5 ± 7.5	**<0.001**
Vitamin A (μg/d)	43 ± 83	10 ± 18	**<0.001**	37 ± 75	17 ± 47	**<0.001**
Thiamine (mg/d)	0.3 ± 0.1	0.3 ± 0.08	0.234	0.4 ± 0.1	0.3 ± 0.1	**<0.001**
Riboflavin (mg/d)	0.3 ± 0.1	0.2 ± 0.1	**0.029**	0.3 ± 0.1	0.2 ± 0.1	**<0.001**
Niacin (mg/d)	5.4 ± 0.9	5.5 ± 0.7	0.472	5.6 ± 0.7	5.1 ± 0.8	**<0.001**
Pyridoxine (mg/d)	0.5 ± 0.1	0.5 ± 0.1	0.731	0.5 ± 0.1	0.4 ± 0.1	**<0.001**
Folate (μg/d)	82 ± 27	81 ± 22	0.762	88 ± 24	70 ± 21	**<0.001**

*Note*: The figures in bold indicate statistically significant *P*‐value.

Abbreviation: IF, indigenous food; SD, standard deviation.

### 3.6 Factors associated with nutrient intake of women with reference to dietary diversity and IF intake

The LMER technique was adopted with villages (*n* = 18) as the random effect. FADI was associated with educational status of women who had education till secondary level and above (*P* = 0.006). Similarly, MDD‐W was also associated with education status of HOH who had education till secondary level and above (*P* = 0.005). No significant relationship was observed between other independent variables. Despite this relationship, while modelling, FADI, MDD‐W and education of both women and HOH were considered as independent variables. Variance inflation factor (VIF) indicated no multicollinearity. However, a significant difference in FADI was observed between various levels of access to food (*P* < 0.001). FADI scores among HHs accessing more than three food sources were significantly different from HHs accessing lower number of food sources. Considering the inter dependence of the two, only FADI was considered while modelling.

In the present study, education status, FADI, MDD‐W and HH wealth index were considered to confound the relationship between IF consumption status and each nutrient intake. Taking into account the confounding effect of these factors, the adjusted findings obtained using LMER are presented in Table 4. Estimates in Table 4 are presented in terms of percentage change in response variable per unit change in the independent variable.

**TABLE 4:** Linear mixed effects regression results of each nutrient intake with dietary diversity and indigenous food consumption adjusting for other characteristics among Sauria Paharia tribal community, Jharkhand, India (*N* = 204)

Characteristics			Energy	Protein	Fat	Vitamin A	Vitamin C	Folate	Vitamin B6	Niacin	Riboflavin	Thiamine	Iron	Calcium	Zinc
IF nonconsumer^a^		Percent change	1.4	1.7	2.0	**−42.6**	1.9	1.3	0.0	2.8	−6.3	**6.6**	2.5	**−10.1**	**4.3**
		95%CI (lower, upper)	(−0.7, 3.6)	(−1.0, 4.7)	(−0.4, 4.6)	**(−66.8, −2.7)**	(−14.4, 21.7)	(−4.4,7.5)	(−3.6, 3.6)	(−0.8, 6.7)	(−13.2, 1.0)	**(0.1, 13.7)**	(−2.9, 8.3)	**(−15.0, −5.1)**	**(0.3, 8.4)**
MDD‐W ≥ 3^b^		Percent change	**5.4**	**9.0**	**8.3**	**189.8**	**39.2**	**21.2**	**7.9**	**9.3**	**20.3**	**24.7**	**14.0**	**13.2**	**12.2**
		95%CI (lower, upper)	**(3.3, 7.7)**	**(6.0, 12.1)**	**(5.7, 11.1)**	**(69.7, 400.3)**	**(16.9, 67.0)**	**(14.2, 28.5)**	**(4.0, 12.0)**	**(5.2, 13.4)**	**(11.4, 29.8)**	**(17.1, 33.0)**	**(7.9, 20.4)**	**(7.1, 19.7)**	**(7.9, 16.6)**
HH wealth index^c^	Lowest middle quintile	Percent change	−1.7	−1.5	**−4.4**	−19.6	1.3	−1.4	−1.8	−2.9	−3.3	−2.5	−1.8	−0.4	−2.5
		95%CI (lower, upper)	(−4.8, 1.3)	(−5.6, 2.6)	**(−7.9, −0.8)**	(−64.0, 80.4)	(−22.7, 31.7)	(−9.8, 7.6)	(−6.9, 3.7)	(−8.1, 2.6)	(−13.8, 8.2)	(−11.3, 7.0)	(−9.5, 6.5)	(−8.2, 8.0)	(−8.0, 3.3)
	Lower middle quintile	Percent change	−2.5	−2.9	**−6.4**	−47.7	−14.6	−7.9	−3.6	−3.4	−6.7	−2.6	−6.5	**−8.7**	−4.0
		95%CI (lower, upper)	(−5.6, 0.7)	(−7.0, 1.4)	**(−10.0, −2.6)**	(−77.0, 24.9)	(−34.9, 12.6)	(−16.1, 0.9)	(−9.0, 1.9)	(−8.9, 2.2)	(−17.1, 5.1)	(−11.9, 7.4)	(−14.2, 1.8)	**(−16.2, −0.4)**	(−9.7, 1.9)
	Upper middle quintile	Percent change	**−3.4**	−3.0	**−4.7**	−52.8	−13.2	−7.7	−5.5	**−6.6**	−7.7	0.7	0.1	−4.8	−3.1
		95%CI (lower, upper)	**(−6.7, −0.2)**	(−7.2, 1.4)	**(−8.4, −0.9)**	(−79.2, 14.5)	(−34.2, 13.8)	(−16.1, 1.2)	(−11.0, 0)	**(−11.9, −1.1)**	(−18.1, 3.8)	(−9.1, 11.2)	(−8.2, 9.2)	(−12.8, 3.8)	(−9.1, 2.7)
	Upper most quintile	Percent change	−2.5	−0.9	**−6.3**	−24.3	−2.0	0.9	−3.1	−2.7	4.9	−3.9	2.4	−2.3	1.2
		95%CI (lower, upper)	(−5.8, 0.7)	(−5.3, 3.5)	**(−10.0, −2.5)**	(−66.8, 84.8)	(−25.2, 29.7)	(−8.1, 10.8)	(−8.7, 2.4)	(−8.3, 3.0)	(−6.8, 18.3)	(−13.3, 6.0)	(−6.0, 11.7)	(−10.5, 6.6)	(−4.8, 7.6)
Education level of women, primary and below^d^		Percent change	−2.2	−2.3	−2.0	−0.9	−0.2	−5.4	−3.0	−4.1	**−10.6**	−3.4	−6.7	−3.6	−4.9
		95%CI (lower, upper)	(−5.0, 0.6)	(−5.9, 1.4)	(−5.2, 1.3)	(−52.5, 103.0)	(−21.3, 27.1)	(−12.5, 2.4)	(−7.6, 1.8)	(−8.8, 0.7)	**(−19.3, −0.9)**	(−11.3, 5.0)	(−13.3, 0.4)	(−10.5, 3.7)	(−9.7, 0.2)
Education level of women,secondary and above^e^		Percent change	−3.3	−4.1	−1.6	−30.8	−6.5	**−14.4**	−5.4	−5.8	**−18.6**	−5.2	−6.9	−8.2	**−8.4**
		95%CI (lower, upper)	(−7.4, 0.9)	(−9.4, 1.5)	(−6.5, 3.6)	(−77.3, 120.6)	(−36.7, 32.6)	**(−24.2, −3.3)**	(−12.2, 1.9)	(−12.7, 1.6)	**(−30.4, −4.9)**	(−16.7, 7.8)	(−16.9, 4.0)	(−18.1, 2.6)	**(−15.5, −0.8)**
Education level of HOH, primary and below^d^		Percent change	−0.2	−1.3	0.3	−7.5	2.0	0.5	−2.4	0.3	−1.7	−7.1	−3.2	4.6	−4.3
		95%CI (lower, upper)	(−5.6, 5.3)	(−10.1, 8.3)	(−10.6, 12.3)	(−73.4, 225.4)	(−28.1, 45.6)	(−18.6, 24.2)	(−11.8, 7.8)	(−8.0, 9.4)	(−17.5, 17.2)	(−23.4, 12.7)	(−20.6, 18.2)	(−14.2, 27.8)	(−14.4, 6.8)
Education level of HOH, secondary and above^e^		Percent change	1.1	0.7	2.5	−47.1	14.7	−4.1	0.3	2.5	−2.9	1.8	−3.0	3.7	−2.1
		95%CI (lower, upper)	(−4.2, 6.8)	(−6.9, 9.3)	(−5.2, 11.0)	(−85.4, 90.4)	(−22.2, 68.2)	(−19.4, 14.2)	(−8.8, 10.4)	(−6.2, 12.1)	(−14.5, 23.6)	(−14.6, 21.4)	(−18.1, 14.2)	(−12.4, 22.1)	(−11.8, 8.5)
FADI		Percent change	4.4	7.8	−5.4	−33.0	−16.3	16.4	1.8	5.7	28.3	10.0	6.6	−2.3	10.5
		95%CI (lower, upper)	(−2.9, 12.2)	(−2.2, 18.8)	(−13.4, 3.3)	(−89.0, 325.0)	(−53.3, 53.1)	(−5.4, 43.0)	(−10.2, 15.3)	(−6.9, 19.7)	(−1.0, 65.9)	(−11.8, 36.6)	(−12.0, 28.9)	(−19.5, 18.3)	(−3.2, 26.2)
ICC			**0.298**	**0.495**	**0.097**	**0.226**	**0.142**	**0.555**	**0.047**	**0.222**	**0.213**	**0.422**	**0.564**	**0.564**	**0.363**

*Note*: Bold numbers indicate significance.

Abbreviations: CI, confidence intervals; FADI, Food Accessed Diversity Index; HOH: head of the household; ICC, intraclass correlation coefficient; IF, indigenous food.

^a^ IF Consumers.

^b^ MDD‐W < 3.

^c^ HH wealth index lowest quintile.

^d^ Above primary level of education.

^e^ Below secondary level of education.

IF consumption was observed to be significantly associated with higher vitamin A and calcium intake after controlling for MDD‐W and other factors in the model (Table 4). An IF consumer was expected to consume 42.6% more of Vitamin A and 10.1% more of calcium than non‐IF consumer. MDD‐W was observed to be significantly associated with all the nutrients after adjusting for IF consumption and other factors in the model.

**FIGURE 2:**
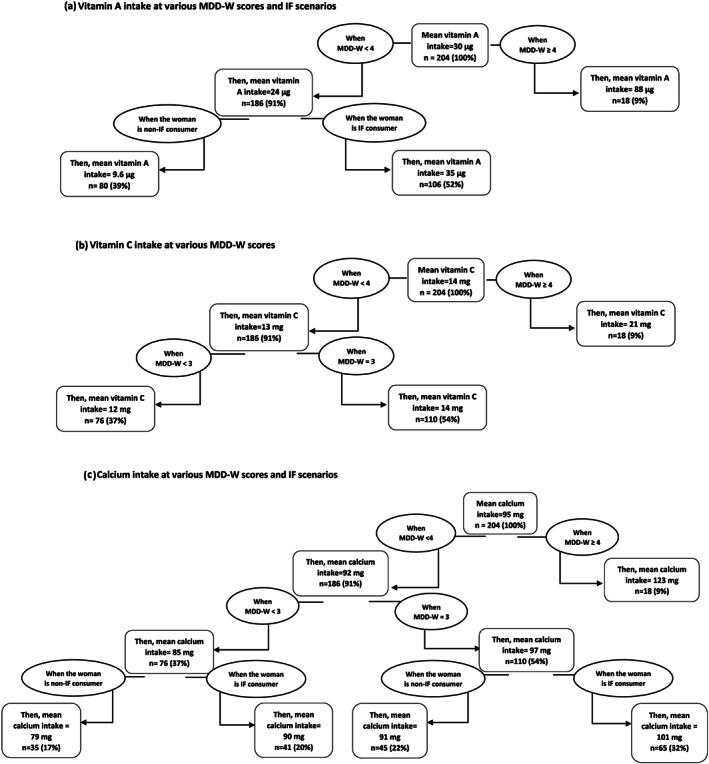
(a–c) Decision trees for micronutrients (values presented are approximate)

Decision trees were developed to perform an objective assessment of various micronutrient consumption at different scenarios of MDD‐W score and IF consumption status among the study population (Figure 2). These assessments revealed 1.3 to 2.9 times higher consumption of different nutrients among the small proportion of women with MDD‐W above or equal to 3 or 4 compared to those with MDD‐W less than 3. For example, though the mean vitamin A intake of the study population was 30 μg/day (considerably lower than EAR), those who had a MDD‐W of more than or equal to 4 had mean intake of 88 μg/day (only 9% of women), whereas those with MDD‐W less than 4 had mean intake of 24 μg. IF consumption among those with MDD‐W lower than 4 improved the vitamin A intake to 35 μg/day (52% of women). Similarly, the mean intake of vitamin C was also low in the study population (14 mg/day). However, the intake improved to 21 mg/day among those who had MDD‐W score of more than or equal to 4 (only 9% of the population). The mean calcium intake of the study women was 95 mg/day. However, women with MDD‐W of more than or equal to 4 had a calcium intake of 123 mg/day. Among women with low MDD‐W (equal to 3), the calcium intake among IF consumers (101 mg/day) was observed to be higher as compared to non‐IF consumers (91 mg/day). IF consumption also improved calcium intake of women with MDD‐W score of less than 3 with an intake of 90 mg compared to non‐consumers with an intake of 79 mg.

### 3.7 CED in women

Based on anthropometric assessment, about 41% women had various degrees of CED, with about 13.4% women falling in the category of CED III (<16 kg/m^2^). CED was, however, not associated with calorie intake (*P* = 0.422; Table 5).

**TABLE 5:** BMI classification of the women of reproductive age (15–49 years), Sauria Paharia tribal community of Jharkhand, India (*n* = 201)

BMI classification in Sauria Paharia women				
Adult subgroup/BMI classification	Underweight (<18.5 kg/m^2^) *n* (%)	Increasing but acceptable risk^a^ (BMI = 18.5–23.0 kg/m^2^) *n* (%)	Increased risk^a^ (BMI = 23.0–27.5 kg/m^2^) *n* (%)	High risk^a^ (BMI > 27.5 kg/m^2^) *n* (%)
Women (15–49 years) *n* = 201	83 (41.3)	107 (53.2)	10 (5)	1 (0.5)

Various grades of CED classification			
Adult subgroup/BMI classification	CED I (17–18.4 kg/m^2^) *n* (%)	CED II (16–16.9 kg/m^2^) *n* (%)	CED III (< 16 kg/m^2^) n (%)
Women (15–49 years) *n* = 83	41 (49.3)	31 (37.3)	11 (13.4)

Abbreviation: BMI, body mass index; CED, chronic energy deficiency.

^a^ The term risk here denotes risk of overweight or obesity.

## 4 DISCUSSION

The present study explored the agroforestry diversity and dietary intake in the context of IF consumption in one of the PVTGs of India, the Sauria Paharias of Jharkhand. We found poor dietary diversity among women of reproductive age, a high prevalence of CED and inadequate consumption of nearly all macronutrients and micronutrients examined. The access to agroforestry diversity as reflected in FADI, despite collective information on several IF sources listed in the food frequency questionnaire, was poor. Women with higher dietary diversity score (MDD‐W) had higher intake of energy, protein, fat, iron, calcium, zinc, B vitamins, vitamin A and vitamin C; also, IF consumers had higher intakes of calcium and vitamin A.

Majority of head of the Sauria Paharia HHs were illiterate and were mainly engaged as agriculturists. The agricultural practices included both traditional farming on the hills, that is, Kurwa as well as farming on agricultural land. Similar profile of this tribal community has been documented from other states also (Kumar & Sinha, 2016; Satyam, 2017). We evaluated the agroforestry diversity in the context of access to food sources by developing an index called the Food Access Diversity Index (FADI), which is adapted from CDI (Michler & Josephson, 2017). We have added a new dimension to the CDI for a more robust view of foods produced and food sources accessed contextual to our study population. This crucial new dimension included sources of food collected and gathered from forests, water bodies apart from cultivated foods. A low FADI score was observed in the study reflecting poor access diversity. This was despite the collective information possessed by the community as demonstrated in the food frequency questionnaire, which included a wide variety of IFs consumed historically. The low FADI could probably be attributed to an overall limited variety of foods accessed at the village and HH level, inter‐HH variability within the village and restricted agricultural diversity. Studies have highlighted educational status of family members as one of the factors influencing the inter HH variability within the village, as better educated people would have more skills and information for accessing and maintaining different varieties of crops and livestock (Di Falco, Bezabih, & Yesuf, 2010; Kissoly, Faße, & Grote, 2018). Other factors reported include socio‐demographic status of the HH, factors associated with climate change like reduced diversity in the forest due to less rain or long summers or water bodies drying up thereby reducing availability and access to fishes and other water animals, promotion of mono crops as part of food policy and shift towards nonagricultural activities (Dhankher & Foyer, 2018; Di Falco et al., 2010; Kissoly et al., 2018; Scheffers et al., 2016; K. Sibhatu, Krishna, & Qaim, 2015).

This poor accessibility was further reflected in the inadequate dietary diversity and nutritional intake among Sauria Paharia women. While we did not find any association between poor MDD‐W with HH wealth index, others have found socio‐economic status, food preferences, lack of time and high opportunity cost for accessing traditional food sources like forests among others as factors affecting consumption of diverse foods (Brashares, Golden, Weinbaum, Barrett, & Okello, 2011). Studies have also cited poor access to land, large family size and lack of knowledge about dietary diversity as the possible factors for poor dietary diversity among women (Powell et al., 2015; Powell, Bezner Kerr, Young, & Johns, 2017). The lack of diversity and overall poor socio‐economic profile of the study population was echoed in the poor nutritional status (41% underweight and poor nutrient intake) of the women, a finding similar to previous studies in tribal communities of India from Jharkhand and other states (Das & Bose, 2015; Ghosh‐Jerath et al., 2016, 2018). Other studies on women from resource poor settings in India and other Asian countries have also reported inadequate energy and micronutrient intake to varying extent. However, our study reports extraordinarily lower levels of nutrient intakes reflecting severe nutritional deprivation. We must also consider that the methodology used to estimate nutrient intake in these studies is not uniform and some have not reported usual intakes as we have (Martin‐Prevel et al., 2017; Pathak, Kapil, Kapoor, Dwivedi, & Singh, 2003; Thankachan, Muthayya, Walczyk, Kurpad, & Hurrell, 2007; Torheim, Ferguson, Penrose, & Arimond, 2010).

Although many indigenous varieties of cereals, GLVs, roots and tubers, other vegetables and fruits were known and potentially available, the access to these foods and frequency of their consumption was low. A typical day's diet for a large majority included rice and some kind of roots and tubers and/or GLVs, a pattern also reported for other tribal communities of Jharkhand and other states (Ghosh‐Jerath et al., 2016, 2018; Thirumani Devi & Sindhuja, 2015).

The energy and protein intake of women was comparatively lower than intakes reported in studies conducted in Santhal and Oraon tribes residing in the state of Jharkhand (Ghosh‐Jerath et al., 2016, 2018). The Sauria Paharia community is considered as PVTG owing to poor development and nutritional indicators and access to health services (Jamwal, 2019; Lal, 2019; MHN Report, 2014). The micronutrient consumption of the community was, however, comparable to that observed in other tribal groups in the region (Ghosh‐Jerath et al., 2016, 2018). Despite an overall low median MDD‐W score, a score of equal to or above 3 was independently associated with higher intake of macronutrients and micronutrients. Moreover, women consuming IFs in the recall period had significantly higher intakes of calcium, riboflavin and vitamin A. This association remained significant for calcium and vitamin A even when adjusted for HH wealth index, FADI and other factors. This was despite the fact that the absolute amount of some of the IFs consumed was quite low. This could be because IFs like GLVs were exceptionally rich in specific nutrients and thus contributed to substantial increase in the nutrient intake even when consumed in low amounts. Previous studies on Santhal and Oraon tribal women also demonstrated a similar outcome of higher mineral intake of iron and calcium upon consumption of IFs (Ghosh‐Jerath et al., 2016, 2018). Several studies in South America and Africa have documented contribution of IFs towards micronutrient intake. These studies reported better intakes of vitamin A, vitamin D and iron among participants who consumed traditional foods (Bersamin, Zidenberg‐Cherr, Stern, & Luick, 2007; Kasimba, Covic, Motswagole, Laubscher, & Claasen, 2019). A study conducted in Peru found that consumption of wild and diverse foods was linked with higher consumption of protein, fibre, iron, calcium, thiamine, riboflavin and vitamin A (Bennett, 2000; Roche, Creed‐Kanashiro, Tuesta, & Kuhnlein, 2008). In developing countries, bush meats and fishes are reported to provide 20% of protein, whereas the IFs in the Gambia have been shown to be important sources of calcium and phosphorus among women (Bennett, 2000; Prentice et al., 1993).

Higher MDD‐W scores in our study were associated with better micronutrient intake. Other studies in low‐income settings from developing countries have also shown poor energy intake and less diverse diets contributing to poor micronutrient intake among women (Arsenault et al., 2013; Henjum et al., 2015). This is crucial from the point of view of developing evidence‐based recommendations and exploring the feasibility and extent of improving nutrient intakes at the cost of improved diversity and IF consumption. Our quantitative estimates using decision trees clearly showed considerable increase in micronutrient consumption upon raising the MDD‐W score or improving IF consumption.

Interventions on improving IF production and consumption have been tried in various developing countries. Strategies include kitchen gardens where indigenous varieties are grown, agricultural interventions for conservation and use of indigenous crop varieties, training and education of food retailers, school feeding programmes, traditional food fairs, among others (‘Homestead food production in Asia: Biodiversity for Food and Nutrition,’; ‘Livestock and diets: Biodiversity for Food and Nutrition’; ‘Students in Nature, in the Garden and in the Kitchen: Biodiversity for Food and Nutrition’). Many of these strategies were well received by the consumers and retailers. Further, they were shown to improve nutrient intake and nutritional status of the vulnerable sections of the community. The current study findings highlight the possibility of improving nutrient intake through incorporation of indigenous varieties of foods in daily diets and improving food access diversity by using multiple sources of food from the food environments. These can be piloted as feasible interventions for tribal communities of Jharkhand. Further, conservation and use of indigenous varieties and strategies for promoting kitchen gardens for growing IFs can be active areas of research.

### 4.1 Limitations

(i) Nutrient intake was calculated using a software based on Indian food composition tables (IFCT) (Longvah et al., 2017) that provides nutritive value of raw Indian foods. The software does not incorporate nutrient retention factors that are the percent adjustments in the nutrients that account for the effect of cooking on nutrient content. (ii) Out of 246 HHs approached, we could complete only 204 dietary surveys. A 17% nonresponse rate was attributed to the locked HHs because of the ongoing sowing season. However, our desirable sample size was 194. (iii) The 24 DR on women documented in this manuscript did not consider seasonal variations, which are also likely to impact the nutrient intake. (iv) We did not assess B_12_ intake in study population, as majority of the women (except 12) did not report consuming any dietary sources of vitamin B_12_ in their diets; further, there is no information on B_12_ content of foods in the IFCT (Longvah et al., 2017). (v) Since the FFQ list was long, we expect reporting and recall bias, and there could also be recall bias while we used 24‐HDR method. (vi) Despite using food recall kit and a portion size estimation flip book, we do expect some level of portion size estimation error in the recalls. (vii) The FADI explored the foods accessed from the natural sources and did not consider the market foods as the purpose of this index was to explore the access to natural IF sources within these communities. (viii) Owing to the cross‐sectional nature of the design, our study presents only associations, and no causal inference can be drawn from the findings. It is also possible that there could be unmeasured sources of confounding in this study.

## 5 CONCLUSION

The Sauria Paharia community of Jharkhand had collective information on several IF sources but was not optimally accessing their diverse agroforestry system. This was reflected in their poor dietary intake and nutritional status. However, a small proportion of this population with higher MDD‐W scores and higher consumption of IFs showed better nutritional intakes. Strategies to promote better access to IFs like promoting production of IFs, information education and communication on nutritive value of IFs and ways of incorporating these in daily diets, school feeding programmes, supplementary feeding programmes and public food distribution system in this community have the potential to complement ongoing nutrition interventions and improve nutritional status of women.

## CONFLICTS OF INTEREST

The authors declare that they have no conflicts of interest.

## CONTRIBUTIONS

S. G. J. and A. S. conceived and designed the study with overall supervision from J. F. and G. G. S. G. J., R. K. and A. S. supervised the entire data collection process. The data analysis was led by N. with assistance from R.K. S. G. J., R. K. and A. S. prepared the first draft of the manuscript. S. D. critiqued and modified the draft. All authors contributed to critique and modification of the manuscript, read and approved the final version. S. G. J. had final responsibility for the decision to submit for publication.

## Supporting information

Table S1: IFs with edible parts and their taxonomic classification
