# Supplementary material for: Agroforestry diversity, indigenous food consumption and nutritional outcomes in Sauria Paharia tribal women of Jharkhand, India
Source: Matern Child Nutr. 2020 Jul 27;17(1):e13052. doi: 10.1111/mcn.13052 (PMC7729651; doi:10.1111/mcn.13052)
Supplement: Supplementary file 1 — Table S1: IFs with edible parts and their taxonomic classification [file MCN-17-e13052-s001.docx]

**Supplementary Table 1: IFs with edible parts and their taxonomic classification**

| **S No.** | **Local Name** | **Common name** | **Scientific name** | **Part Consumed** | **Food group** |
| --- | --- | --- | --- | --- | --- |
| 1-11. | Swarna, Bhadai , Sarda, Arwa , Sarap , Jesori, Bahyar, Bangla Bhat, Hariya, Banagal, Lal Dhan | Varieties of Rice | *Oryza sativa* | Grain | Cereals |
|  | Makai/Gangi (Desi) | Maize | *Zea mays* | Cob | Cereals |
|  | Bajra/Shishua (Desi) | Pearl millet | *Pennisetum typhoideum* | Kernel | Cereals |
|  | Kodo/Mandua (Desi) | Finger millet | *Eleusine coracana* | Grain | Cereals |
|  | Jowar (Desi) | Sorghum | *Sorghum vulgare* | Grain | Cereals |
|  | Gondli***** | NA | NA | Grain | Cereals |
|  | Jatta usro/Ghangra | Cowpea, brown | *Vigna catjang* | Seed and vegetable | Pulses; other vegetables |
|  | Eso Usro | Cowpea, white | *Dolichos catjang* | Seed | Pulses |
|  | Kakro/Suthro/Suthri | Rice bean | *Phaseolus calcaratus* | Seed | Pulses |
|  | Kurthi | Horse Gram, whole | *Dolichos biflorus* | Seed | Pulses |
|  | Khesari ( Desi) | Khesari | *Lathyrus sativus* | Seed and leaves | Pulses; GLVs |
|  | Kusa | NA | *Mucuna pruriens* | Seed | Pulses |
|  | Makedi | Colocasia leaves | *Colocasia antiquorum* | Leaves and root | GLVs; Roots and tubers |
|  | Komo | Koinaar leaves | *Bauhinia purpurea* L. | Leaves | GLVs |
|  | Daav Ghasi | Kantha leaves | *Euphorbia granulate* | Leaves | GLVs |
|  | Lol Ghasi | Bottlegourd leaves | *Lagenaria vulgaris* | Leaves | GLVs |
|  | Tisso Ghasi | Mata leaves | *Antidesma diandrum* | Leaves and fruit | GLVs; fruits |
|  | Chilo Ghasi/Kodgo | Sinduar leaves | *Celosia argentia* | Leaves | GLVs |
|  | Aloo Ghasi | Potato greens | *Solanum tuberosum* | Leaves | GLVs |
|  | Sanjhori | Drumstick | *Moringa oleifera* | Leaves and flower | GLVs; other vegetables |
|  | Adro Ghasi/Margi adro | Amaranth leaves | *Amaranthus spinosus* | Leaves | GLVs |
|  | Gochi Ghasi | Ponnaganni | *Alternanthera sessilis* | Leaves | GLVs |
|  | Berbayo Ghasi | Kena leaves | *Commelina benghalensis* | Leaves | GLVs |
|  | Kondi Ghasi | Dhurup leaves | *Leucas lavandulifolia* Sm*.* | Leaves | GLVs |
|  | Boot Ghasi | Bengal gram leaves | *Cicer arietinum* | Leaves | GLVs |
|  | Naolo Ghasi | NA | *Trianthema portulacacstrum* L | Leaves | GLVs |
|  | Pakkedi | Banyan leaves | *Ficus bengalenis* | Leaves and fruit | GLVs; fruits |
|  | Junjuni/Amadro | Susni leaves | *Marsilea minuta* | Leaves | GLVs |
|  | Nasni Ghasi | Garlic leaves | *Allium sativum* | Leaves | GLVs |
|  | Pondka Saag | Malabar spinach | *Basella rubra* | Leaves | GLVs |
|  | Zaraael | Ziruli leaves | *Indigofera cassioides* | Leaves and flower | GLVs; other vegetables |
|  | Sonpu | NA | *Crotolaria juncea* | Leaves and flower | GLVs; other vegetables |
|  | Chiniya Saag | NA | *Brassica campestris* | Leaves | GLVs |
| 44-57. | Gobero Adro, Pusre Adro, Gutni, Chiroti Saag, Acchadro, Aradiyo Ghasi, Ursudi Ghasi, Jonya Ghasi, Kannasedi saag, Bodo Ghasi, Dababotri Ghasi, Kotua Saag, Madhari Saag, Manaadro Saag***** | Unidentified folk species of GLVs | | Leaves | GLVs |
|  | Bir Karela | Bittergourd | *Momordica charantia* | Vegetable | Other vegetables |
|  | Simbi | Field beans, tender | *Dolichos lablab* | Vegetable | Other vegetables |
|  | Jhingli (Desi) | Ridgegourd | *Luffa acutangula* | Vegetable | Other vegetables |
|  | Maas ardo /Karu/Baans | Bamboo tender | *Bambusa vulgaris* | Vegetable | Other vegetables |
|  | Kokri | Spinegourd | *Momordica dioica* | Vegetable | Other vegetables |
|  | Kundri | Kovai | *Coccinia cordifolia* | Vegetable | Other vegetables |
|  | Kachna Phool | Kachnar flower | *Bauhinia variegata* L. | Flower | Other vegetables |
|  | Beralli | NA | *Dioscorea* spp. | Vegetable and root | Other vegetables; Roots and tubers |
|  | Pinra/ Pindra | NA | *Flacourita indica* | Vegetable and root | Other vegetables; Roots and tubers |
|  | Zarkunda | Ashgourd | *Benincasa hispida* | Vegetable | Other vegetables |
|  | Misrikand | Yam beans | *Pachyrhizus erosus* | Root | Roots and tubers |
|  | Lal Aloo | Red Potato | *Solanum tuberosum* | Root | Roots and tubers |
|  | Nappe/Nappa | NA | *Dioscorea pentaphylla* L. | Root | Roots and tubers |
|  | Singla | Ole | *Amorphophallus paeoniifolius* | Root | Roots and tubers |
| 72-87. | Jattali, Porniali, Churka/Churke, Taalko, Chalangan/ Chalango, Pandgo/Pangdro,Chambiyali, Gumalli, Alli, Isaha Alli, Aeli, Gomo, Chalko,Igzol,Panne, Keso ***** | Unidentified folk species of Roots and tubers | | Root | Roots and tubers |
|  | Ambad Pupu | Ambada | *Spondias mangifera* | Fruit | Fruits |
|  | Madgi | Mahua | *Madhuca indica* | Fruit | Fruits |
|  | Kero/Keero Toso | Marking nut (kernel) | *Semecarpus anacardium* | Fruit | Fruits |
|  | Ilkarpu/Ber  **Table**  (Continued) | Zizyphus | *Zizyphus jujube* | Fruit | Fruits |
|  | Telo/Kenda/Kaanda/Kendu | Tumki | *Diospyros melanoxylon* | Fruit | Fruits |
|  | Talmi | Palmyra fruit, ripe | *Borassus flabellifer* | Fruit | Fruits |
|  | Pusra | Kusum | *Scheleichera oleosa* | Fruit | Fruits |
|  | Dahu/Tisgo Chagzo | NA | *Artocarpus lakoocha* | Fruit | Fruits |
|  | Piyaara | NA | *Buchanania lanazan* Spr. | Fruit | Fruits |
|  | Haani | NA | *Ficus exasperata* Vahl | Fruit | Fruits |
|  | Bel/Otte | Wood apple | *Aegle marmelos* | Fruit | Fruits |
|  | Dumari/Dumaari/Dungri | NA | *Ficus glomerata* | Fruit | Fruits |
| 100-104. | Zara Aeli,Anni,Kaisge,Kaita,Dhela***** | Unidentified folk species of fruits | | Fruit | Fruits |
|  | Singhi Machhli | Singhi | *Saccobranchus fossilis* | Meat | Flesh foods |
|  | Potha/Pothi Machhli | Puti | *Burbus* spp*.* | Meat | Flesh foods |
|  | Ghongri | Snail | *Pila globoasa* | Meat | Flesh foods |
|  | Maako/Jhinuk | Mussels | *Margaritifera margaritifera* | Meat | Flesh foods |
|  | Magur/Mangri Machhli | Walking catfish | *Clarias batrachus* | Meat | Flesh foods |
|  | Boari Machhli | Wallago | *Wallago attu* | Meat | Flesh foods |
|  | Gacchi Machhli | Freshwater Eel | *Anguilla anguilla* | Meat | Flesh foods |
|  | Tengra/Tonger Machhli | Catfish | *Mystus vittatus* | Meat | Flesh foods |
|  | Silong Machhli | Silhan | *Silonia silondia* | Meat | Flesh foods |
|  | Gadai/ Gowari Machhli | Snake head fish | *Channa punctate* | Meat | Flesh foods |
|  | Chala/Chalgo Machhli | Silver razor belly minnow | *Salmostoma acinaces* | Meat | Flesh foods |
|  | Baale Machhli | Bele | *Glossogobius giuris* | Meat | Flesh foods |
| 117-129. | Zimali, Eherchali, Chachara, Chuchi, Mustura, Gotti, Doke, Mitra, Erke, Banjakudi, , Chete , Chatarkati, Jambuchett Machhli***** | Varieties of fish | | Meat | Flesh foods |
| 130. | Chetado ka anda | Eggs of red ants | *Aceophylla smaragdina* | Meat | Flesh foods |
| 131. | Gilhari ka maas | Squirrel meat | *Sciuridae* | Meat | Flesh foods |
| 132. | Moosa | Field's rat | *Rattus argentiventer* | Meat | Flesh foods |
| 133. | Chuwa | Peacock | *Pavo cristatus* | Meat | Flesh foods |
| 134. | Mahalo/Mahala | Wild cat | *Felis catus* | Meat | Flesh foods |
| 135. | Jangli suar/Kissu | Pig | *Sus scrofa* | Meat | Flesh foods |
| 136. | Kissa/Chitru | Porcupine | *Erethizon dorsatum* | Meat | Flesh foods |
| 137. | Jangli murgi | Fowl | *Galloanserae* | Meat | Flesh foods |
| 138. | Teetar | Partridge | *Grey francolin* | Meat | Flesh foods |
| 139. | Pervan | Pigeon | *Columba livia domestica* | Meat | Flesh foods |
| 140. | Bater | Quail | *Coturnix coturnix* | Meat | Flesh foods |
| 141. | Edru/tota | Parrot | *Psittacine* | Meat | Flesh foods |
| 142-147. | Tirikado (Chidiya), Pura (Chidiya), Tenga, Kafo, Tura, Oda ***** | Unidentified folk species of birds | | Meat | Flesh foods |
| 148-150. | Haubudu, Teni, Isge***** | Unidentified folk species of insect | | Honey | Sugar |
| 151-183. | Makko/ Maango, Kero/Aero/Khero, Parango, Baado, Endro/Adro/Edro, Naango, Baansosu, Tele-kuto/Telo-kuti, Kaijo, Takna, Tero, Baalco, Ado, Korho/Orho, Kerusudo, Patanglo/Pattangulo/Putka, Pattodi/ Pittodi, Chaandi, Jambuaajo, Chaariyoni, Gobroosu, Baanipoto, Kuttapuda, Bandho aero, Bagdoto, Jhinganu/Jhingan, Nalo Osu, Baloosu, Mokro/Mokeroosu, Patla aero, Isuno, Jinpro aero, Ganda budi***** | Unidentified folk species of mushrooms | | Upper part of mushroom  (cap )and stem | Mushrooms |

NA: Not available GLV: Green leafy vegetables

***** Taxonomic classification not available
